# *db*/*db* Mice Exhibit Features of Human Type 2 Diabetes That Are Not Present in Weight-Matched C57BL/6J Mice Fed a Western Diet

**DOI:** 10.1155/2017/8503754

**Published:** 2017-09-06

**Authors:** Susan J. Burke, Heidi M. Batdorf, David H. Burk, Robert C. Noland, Adrianna E. Eder, Matthew S. Boulos, Michael D. Karlstad, J. Jason Collier

**Affiliations:** ^1^Laboratory of Immunogenetics, Pennington Biomedical Research Center, Baton Rouge, LA 70808, USA; ^2^Laboratory of Islet Biology and Inflammation, Pennington Biomedical Research Center, Baton Rouge, LA 70808, USA; ^3^Skeletal Muscle Metabolism Laboratory, Pennington Biomedical Research Center, Baton Rouge, LA 70808, USA; ^4^Cell Biology and Bioimaging Core Facility, Pennington Biomedical Research Center, Baton Rouge, LA 70808, USA; ^5^Department of Surgery, Graduate School of Medicine, University of Tennessee Health Science Center, Knoxville, TN 37920, USA

## Abstract

To understand features of human obesity and type 2 diabetes mellitus (T2D) that can be recapitulated in the mouse, we compared C57BL/6J mice fed a Western-style diet (WD) to weight-matched genetically obese leptin receptor-deficient mice (*db/db*). All mice were monitored for changes in body composition, glycemia, and total body mass. To objectively compare diet-induced and genetic models of obesity, tissue analyses were conducted using mice with similar body mass. We found that adipose tissue inflammation was present in both models of obesity. In addition, distinct alterations in metabolic flexibility were evident between WD-fed mice and *db/db* mice. Circulating insulin levels are elevated in each model of obesity, while glucagon was increased only in the *db/db* mice. Although both WD-fed and *db/db* mice exhibited adaptive increases in islet size, the *db/db* mice also displayed augmented islet expression of the dedifferentiation marker Aldh1a3 and reduced nuclear presence of the transcription factor Nkx6.1. Based on the collective results put forth herein, we conclude that *db/db* mice capture key features of human T2D that do not occur in WD-fed C57BL/6J mice of comparable body mass.

## 1. Introduction

Obesity is a major risk factor for insulin resistance, metabolic syndrome, and type 2 diabetes (T2D) [1, 2]. In the human population, body mass index calculations are often used to determine metabolic disease risk [3], with stratifications ranging from nonobese (18.5–24.9 kg/m^2^) to overweight (25–30 kg/m^2^) to obese (>30 kg/m^2^). Different metabolic disturbances and degrees of risk are often detected within each subgroup [3, 4]. Consequently, increasing body mass in the form of excess adiposity is often detrimental to whole body glucose homeostasis due to a chronic low-grade inflammatory condition [5].

On a cellular level, adipocytes play a key role in both fat storage and production of soluble factors that regulate insulin sensitivity [6]. In addition, whether excess intracellular lipid content is stored in adipose tissue or in lean tissues has major implications for disease risk [7, 8]. Storage of lipid in lean tissues is detrimental and produces adverse metabolic outcomes [7]. The chronic low-grade inflammation associated with an overabundance of adipose tissue often leads to endocrine alterations and dysfunction of other tissues, such as the skeletal muscle and liver [5].

During insulin resistance and “prediabetes”, islet *β*-cells typically increase in number via proliferation of the existing cellular population [9–11]. In addition, insulin output is enhanced prior to diabetes onset to compensate for reduced action of the hormone in peripheral tissues (e.g., skeletal muscle and liver). Once a decrease in islet *β*-cell insulin secretion and diminutions in the *β*-cell population or both occur, clinical onset of T2D ensues [12]. Clearly, there are stages of disease progression with earlier metabolic aberrations (e.g., hyperinsulinemia) that ultimately indicate risk of onset of T2D. Hyperinsulinemia and insulin resistance may often go undiagnosed but are important “prediabetic” indicators of future disease risk. Progression to overt diabetes requires a diminution in pancreatic islet *β*-cell mass, *β*-cell function, or both [1, 9, 13].

Pancreatic islet *β*-cells from human subjects with T2D display markers of dedifferentiation. For example, increased abundance of Aldh1a3 and reduced nuclear presence of the transcription factor Nkx6.1 were observed in pancreatic islets from T2D, but not in healthy controls [14]. Nkx6.1 is important for islet *β*-cell development, suppressing expression of the glucagon gene, and for maintenance of adult *β*-cell phenotype [15, 16]. With elevated glucagon levels being a long known feature of uncontrolled diabetes [17], it is not surprising that losses in key transcription factors within islet *β*-cells could lead to dysregulation of overall islet hormone production.

During a comparison of C57BL/6J mice fed a high-fat, sucrose-enriched Western diet (WD) with weight-matched genetically obese leptin receptor-deficient mice (*db/db*), several key findings arose: (1) WD-fed and *db/db* mice accumulate body weight and fat mass over time at rates higher than their respective lean control counterparts but similar to each other. (2) Both WD-fed C57BL/6J mice and *db/db* mice are hyperinsulinemic, but only *db/db* mice are hyperglycemic. (3) Circulating insulin levels are elevated in both models of obesity, but glucagon is increased only in the *db/db* mice. (4) While both WD-fed C57BL/6J mice and *db/db* mice exhibit adaptive increases in islet size, the *db/db* mice also display increased islet expression of the dedifferentiation marker Aldh1a3. (5) There is reduced nuclear presence of the transcription factor Nkx6.1 in *db/db* mice when compared with mice fed the WD. We conclude that *db/db* mice capture key features of human T2D that do not occur in weight-matched WD-fed C57BL/6J mice.

## 2. Methods

### 2.1. Animals and Reagents

Nine-week-old male C57BL/6J (stock number 00664) and 5-week-old male *db*/+ and *db/db* mice (B6.BKS(D)-*Lepr^db^/*J; stock number 00697) were acquired from the Jackson Laboratories (Bar Harbor, Maine). These mice were allowed to acclimate to the photoperiod (12-hour light/12-hour dark) and temperature conditions (22 ± 1°C) for one week prior to the start of the study. C57BL/6J mice were randomized into their respective dietary group assignments based on body weight and blood glucose measurements such that no significant differences existed between groups at baseline. At 10 weeks of age, 3 separate cohorts of mice were started on either control diet (Cat number 98052602; Research Diets Inc., New Brunswick, NJ) or Western diet (Cat number D12079B; Research Diets Inc.) for 4, 12, and 20 weeks. The *db/db* and *db*/+ mice were each given access to Lab Diet 5001 ad libitum throughout the study to represent conditions similar to those used for colony maintenance at the Jackson laboratory. Blood glucose was measured weekly using an Accu-Chek Aviva Plus Glucometer (Roche Diagnostic, Indianapolis, IN). Weekly measurements of body mass and composition (fat, lean, and fluid mass) were assessed by NMR using a Bruker Minispec LF50 Time-Domain NMR system. Upon completion of the study feeding period, animals were sacrificed by CO_2_ asphyxiation and decapitation. Whole pancreata were fixed in 10% neutral-buffered formalin overnight prior to sectioning and embedding. Fat depots, liver, and skeletal muscle were flash frozen in liquid nitrogen and stored at −80°C. Serum was collected from trunk blood or cardiac sticks. Pancreatic islets were isolated as described previously [18]. Relevant animal procedures were approved by the PBRC and the University of Tennessee Institutional Animal Care and Use Committees.

### 2.2. Insulin Tolerance Test, Islet Histology, and Substrate Oxidation

Mice were fasted for 2 h and were injected intraperitoneally with 0.75 units/kg Humulin R insulin as previously described [19]. Islet histology procedures have been described [19, 20]. Briefly, the antibodies used were as follows: insulin (Invitrogen; 18-0067; used at 1 : 800), glucagon (Cell Signaling Technologies; 8233; used at 1 : 400), Aldh1a3 (Novus Biologicals; NBP2-15339; used at 1 : 100), and Nkx6.1 (F55A12; 1 : 200; [21]). All antibodies were incubated overnight at 4°C and detected with either Alexa Fluor secondary conjugation (Alexa 488, 594), except for Nkx6.1, which was detected using a biotin/streptavidin exposure method. Substrate oxidation was measured in the liver, and gastrocnemius muscle homogenates were prepared and used as described previously [22, 23]. Briefly, using [1-^14^C] palmitate (100 *μ*M), both complete (^14^CO_2_) and incomplete (^14^C-acid soluble metabolites (ASMs)) fatty acid oxidation were assessed ± varying doses of unlabeled pyruvate (0, 0.1, 1, and 10 mM) to assess metabolic flexibility.

### 2.3. Total RNA Extraction, cDNA Synthesis, and Real-Time RT-PCR

All procedures have been described previously [24, 25]. Briefly, total RNA was extracted from mouse islets, liver, and epididymal fat depots using an RNeasy Mini Kit (Qiagen). RNA quality and quantity was assessed using a Nanodrop spectrophotometer (Thermo Scientific). cDNA was generated from total RNA using the iScript cDNA synthesis kit (Bio-Rad). Relative mRNA abundance was measured by real-time PCR using the iTaq Universal SYBR Green Supermix (Bio-Rad) on a CFX96 instrument (Bio-Rad). Transcript levels were normalized with the housekeeping gene *Ppia* or Rs9. Primer pairs were designed using the Primer3Plus software.

### 2.4. Serum ELISA

The following kits were used to measure serum factors: Mouse/Rat Leptin Quantikine ELISA kit (Cat number MOB00) from R&D Systems (Minneapolis, MN), Mouse Insulin ELISA kit (Cat number 10-1247-01) and Glucagon ELISA kit (Cat number 10-1271-01) from Mercodia (Uppsala, Sweden), Corticosterone ELISA kit (Cat number ADI-900-097) from Enzo Life Sciences (Farmingdale, NY), and the Mouse Adiponectin ELISA kit (Cat number 80569) from Crystal Chem (Downers Grove, IL). Manufacturer's recommended protocols were used for all serum measurements.

### 2.5. Tissue Acyl Glycerol Measurements

Isolated liver (30 mg) tissue was homogenized in 5% NP-40 solution (300 *μ*L) and heated twice to 95°C to solubilize all acyl glycerol species. Samples were centrifuged at maximum speed for 2 minutes, and supernatant was removed to a clean tube to prevent contamination with insoluble material. The transferred samples were diluted 10-fold in water before proceeding with the detection portion of the assay. Acyl glycerol content was measured using the Triglyceride Determination kit (Cat number TR0100) from Sigma Aldrich according to the manufacturer's directions.

### 2.6. Statistics

The data were evaluated with the GraphPad Prism 6.0 statistical analysis software. Data were analyzed by either one-way ANOVA using a Tukey post hoc, repeated measures ANOVA (for longitudinal measures of blood glucose, body weight, and body composition), two-way repeated measures ANOVA with Bonferroni post hoc test for multiple comparisons (substrate oxidation), or two-tailed Student's *t*-test. Individual *p* values are given in the figure legends.

## 3. Results

### 3.1. C57BL/6J Mice Fed a High-Fat, Sucrose-Enriched Diet Gain Weight but Do Not Develop Hyperglycemia

**Figure 1:**
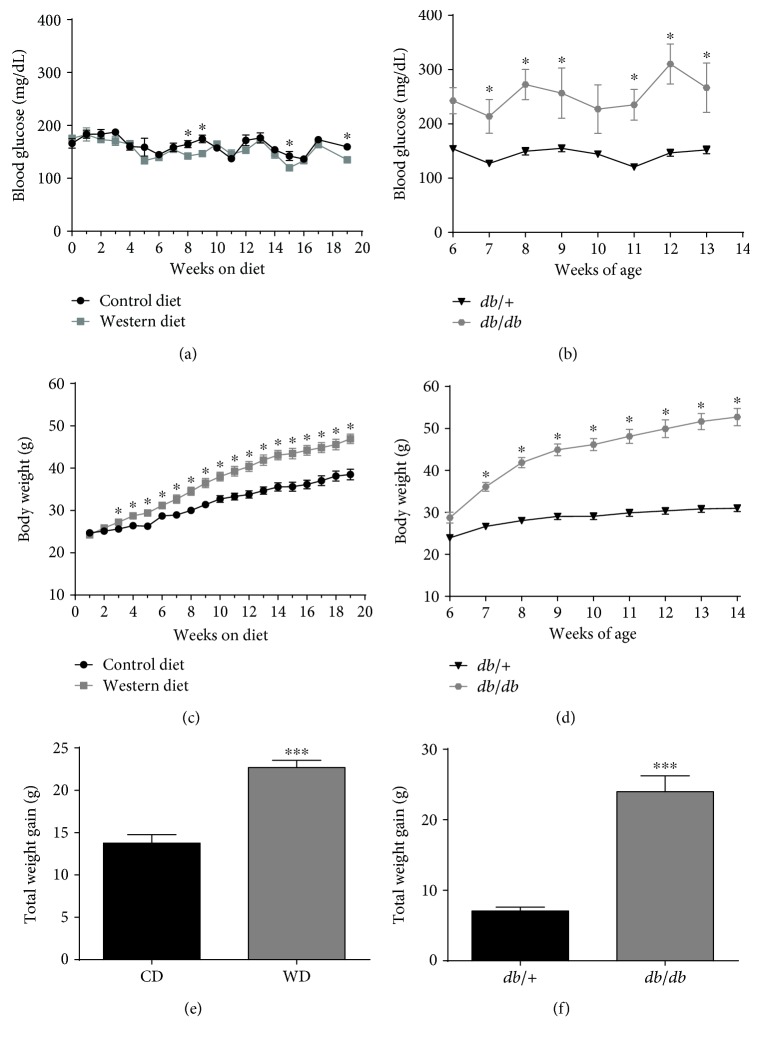
Hyperglycemia develops in genetically driven, but not diet-induced obesity. Blood glucose (a) and body weight (c) in male C57BL/6J mice fed either control or Western diet for 20 weeks. Total weight gain (e) at the end of the 20-week feeding period. Blood glucose (b) and body weight (d) in male *db*/+ and *db*/*db* mice between 6 and 14 weeks of age. Total weight gain by 14 weeks of age (f). For body weight measurements (c and d), *y-*axes are set to the same scale for comparison between study groups. *n* = 8 per group; means ± SEM; ^∗∗∗^*p* < 0.001 versus control; ^∗^*p* < 0.05 versus control by repeated measures ANOVA. CD: control diet; WD: Western diet.

Over the course of 20 weeks, male C57BL/6J mice were fed either a high-fat, high-sucrose Western diet (WD) or sucrose-matched low-fat control diet (CD). Blood glucose remained stable in both groups of mice during this 20-week period (Figure 1(a)). The study conditions were closely monitored so that mice fed the WD could be weight matched with *db/db* mice. The *db/db* mice on the C57BL/6 genetic background, which develop obesity secondary to leptin resistance, display noticeable elevations in blood glucose early in their lifespan (Figure 1(b)). The *db/db* mice were compared to lean littermate heterozygous controls for the leptin receptor mutation (*db*/+). The hyperglycemia in *db/db* mice was maintained throughout the eight-week study period. C57BL/6J mice fed the WD gained 22.7 g of total body mass versus 13.8 g for mice fed the CD (Figures 1(c) and 1(e)). The *db/db* mice were fed ad libitum with a low-fat diet (6% kcal) to mimic conditions commonly used for this strain. On the low-fat diet, the *db*/+ animals gained 7.1 g, while *db/db* mice increased total body weight by 24 g (Figures 1(d) and 1(f)). Therefore, although similar accumulation of body mass was observed in C57BL/6J on a Western-style diet when compared to *db/db* mice on a low-fat diet, there was a noticeable difference in glycemic control between these mouse models of obesity.

### 3.2. *db/db* Mice Accumulate Adipose Tissue on a Low-Fat Diet Similar to That Seen with C57BL/6J Mice Fed a Western Diet

**Figure 2:**
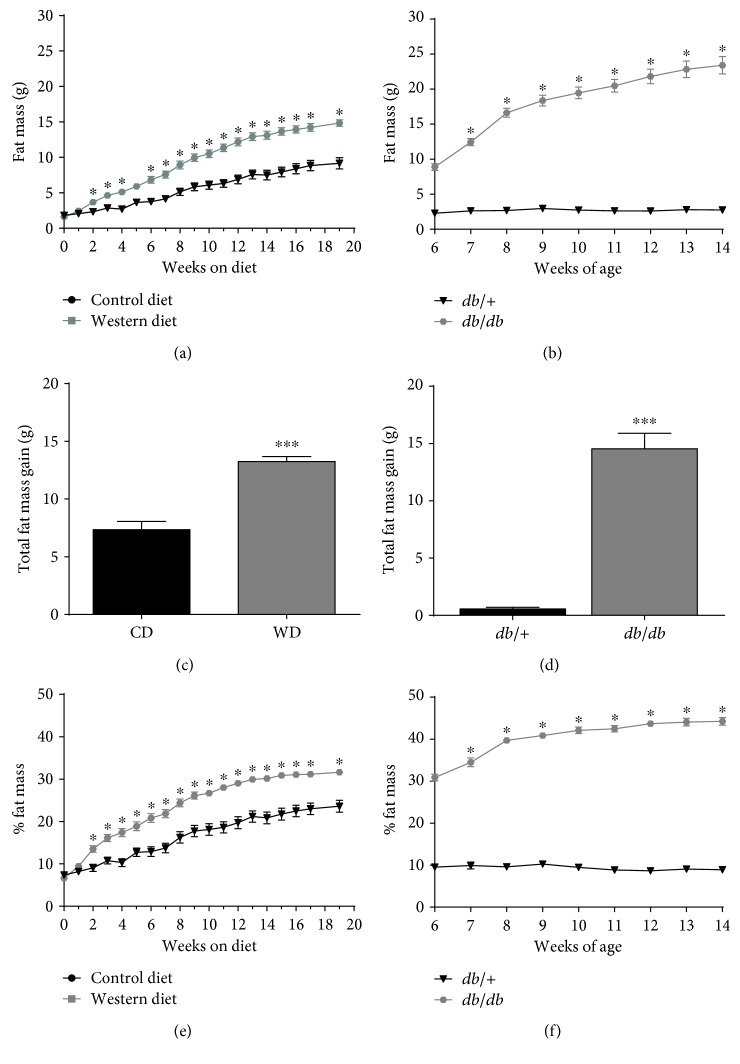
Increased fat mass occurs in both diet-induced and genetically driven forms of obesity. Fat mass (a) and % fat mass (e) in male C57BL/6J mice fed either control or Western diet for 20 weeks. Total fat mass gain (c) at the end of the 20-week feeding period. Fat mass (b) and % fat mass (f) in male *db*/+ and *db*/*db* mice between 6 and 14 weeks of age. Total fat mass gain by 14 weeks of age (d). For fat mass measurements, *y*-axes are set to the same scale for comparison between study groups. *n* = 8 per group; means ± SEM; ^∗∗∗^*p* < 0.001 versus control; ^∗^*p* < 0.05 versus control by repeated measures ANOVA. CD: control diet; WD: Western diet.

**Figure 3:**
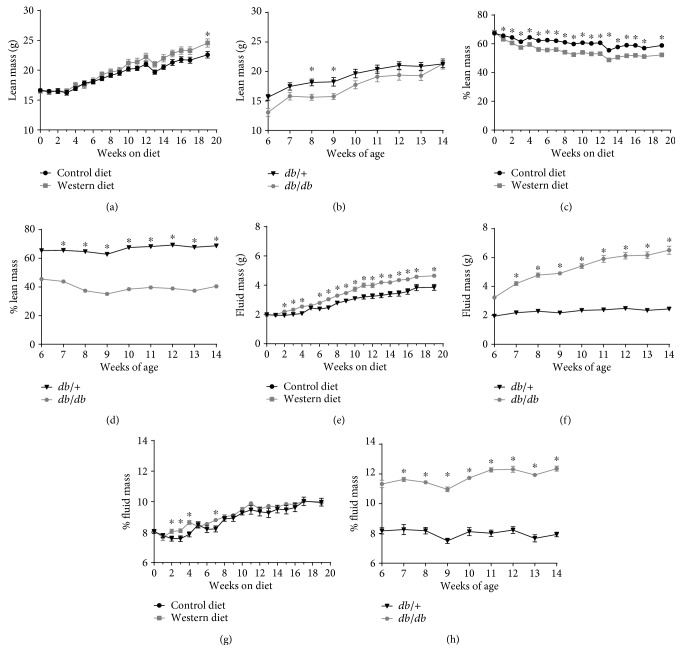
Lean and fluid mass profiles in WD-fed and genetically obese *db/db* mice. Lean mass (a), % lean mass (c), fluid mass (e), and % fluid mass (g) in male C57BL/6J mice fed either control or Western diet for 20 weeks. Lean mass (b), % lean mass (d), fluid mass (f), and % fluid mass (h) in male *db*/+ and *db/db* mice between 6 and 14 weeks of age. *n* = 8 per group; means ± SEM; ^∗^*p* < 0.05 versus control by repeated measures ANOVA.

Because the accrual of adipose tissue (fat mass) impacts systemic metabolic parameters [6], we investigated the accumulation of this tissue depot in both diet-induced and genetic models of obesity. C57BL/6J mice fed the WD displayed consistently elevated fat mass over mice in the CD group (Figure 2(a)). We found that *db/db* mice between the ages of 6 and 14 weeks of age gained a similar amount of fat mass (14.5 g) as the C57BL/6J mice on a WD for 20 weeks (13.25 g; compare Figure 2(c) with Figure 2(d)). However, *db*/+ mice did not accumulate significant amount of fat mass between the ages of 6 and 14 weeks (0.56 g; Figures 2(b) and 2(d)), which we suspect is due to the low amount of fat and sucrose in this diet. When viewed from a perspective of fat mass as a percentage of total body mass, the C57BL/6J mice start out with a relatively low total body fat percentage (~6%) but go over 30% body fat by week 20 of WD feeding (Figure 2(e)). Alternatively, the *db/db* mice start out near 30% total body fat at 6 weeks of age and increased to over 40% at 14 weeks of age (Figure 2(f)). When lean mass was examined, both C57BL/6J mice fed a WD and *db/db* mice display similar increases in total lean mass, although the *db/db* mice had a lower *total percentage* of whole body lean mass (Figure 3). Similar findings were observed when measuring fluid mass (Figure 3).

### 3.3. Elevations in Serum Leptin Are Consistent with Increases in Body Weight and Adiposity in Both Models of Obesity, While Elevated Corticosterone Is Specific to *db*/*db* Mice

**Figure 4:**
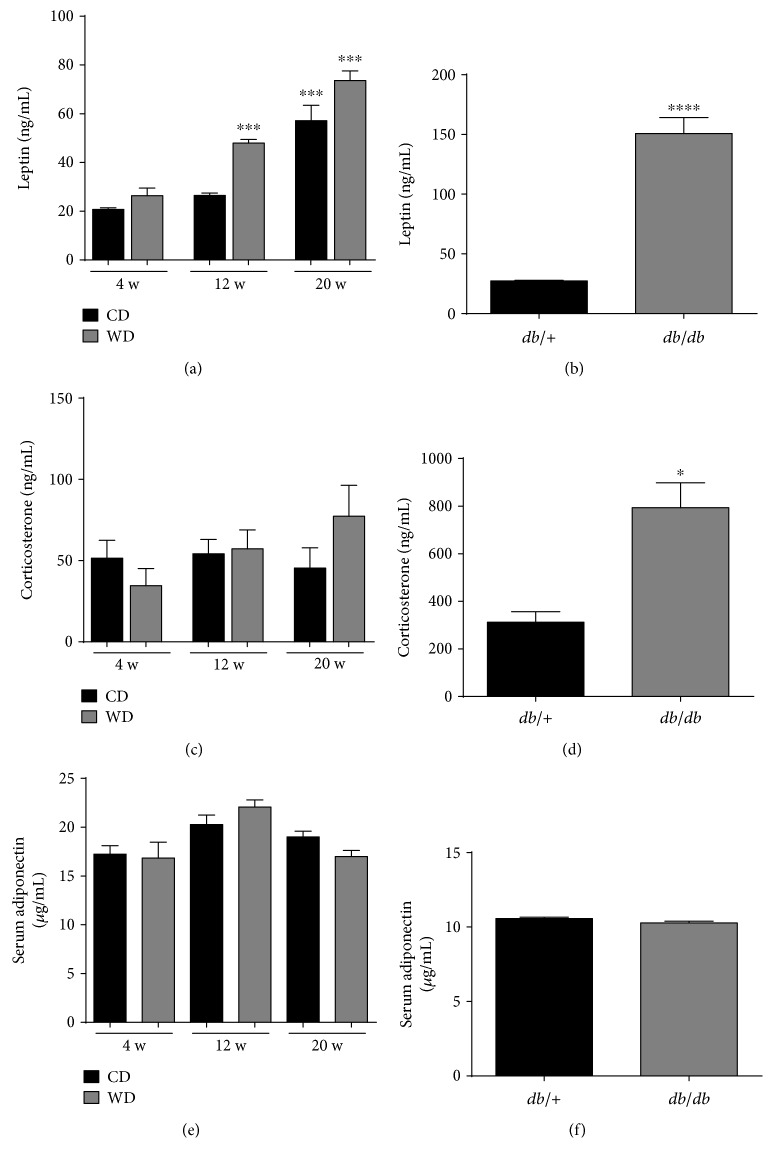
Elevated corticosterone in serum of *db/db* mice but not WD-fed mice. Serum levels of leptin (a, b), corticosterone (c, d), and adiponectin (e, f) in C57BL/6J mice fed either control or Western diet for 4, 12, and 20 weeks (a, c, e) or 14-week-old *db*/+ and *db*/*db* mice (b, d, f). *n* = 8 per group; means ± SEM. ^∗∗∗∗^*p* < 0.0001; ^∗∗∗^*p* < 0.001; ^∗^*p* < 0.05. CD: control diet; WD: Western diet; HFD.

Leptin is a fat cell-derived hormone that has important roles in regulating satiety. Serum leptin clearly increases with body weight in WD-fed mice and is also elevated as the mice get older (Figure 4(a)). In addition, *db/db* mice have elevations in serum leptin over their lean counterparts (Figure 4(b)). By contrast, corticosterone is relatively stable in the serum of WD-fed mice (Figure 4(c)) but elevated in *db/db* mice (Figure 4(d)). Adiponectin, also derived from adipose tissue, was not impacted by body weight, adiposity, or age of the mice (Figures 4(e) and 4(f)).

### 3.4. There Is Clear Evidence of Inflammation in Adipose Tissue from C57BL/6J Mice Fed a WD and in *db*/*db* Mice

**Figure 5:**
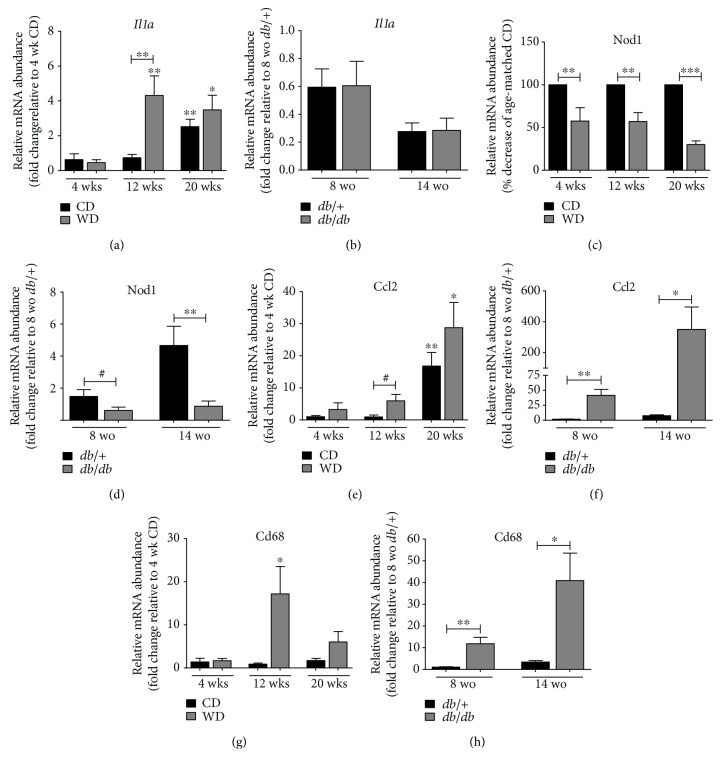
Increased expression of inflammatory markers is evident in epididymal adipose tissue from both genetic and diet-induced obesity. Relative mRNA abundance of the inflammatory genes *Il1a* (a, b), *Nod1* (c, d), *Ccl2* (e, f), and *Cd68* (g, h) in epididymal adipose tissue from either C57BL/6J mice fed either control or Western diet for 4, 12, and 20 weeks (a, c, e, g) or 8- and 14-week-old *db*/+ and *db/db* mice (b, d, f, h). *n* = 8 per group. Data are normalized to the reference gene *Ppia* and are represented as means ± SEM. ^∗∗∗^*p* < 0.001; ^∗∗^*p* < 0.01; ^∗^*p* < 0.05; ^**#**^*p* < 0.1 by one-way ANOVA with Tukey's post hoc analysis. (a), (e), and (g) *p* values versus respective 4-week dietary group unless denoted by bar. CD: control diet; WD: Western diet; wo: weeks old.

With clear increases in fat mass accumulation between WD and *db/db* mice (Figure 2(c) versus Figure 2(d)), we next measured the expression of genes involved in inflammatory responses using isolated epididymal white adipose tissue (eWAT). Expression of the *Il1α* gene, which encodes a proinflammatory cytokine, was strongly upregulated in animals fed WD for 12 weeks relative to mice eating the CD (Figure 5(a)). By 20 weeks, mice on the control diet were also displaying elevations in this cytokine (Figure 5(a)); in our view, this increase in inflammation in control mice is most likely due to aging. Notably, expression of IL-1*α* was not significantly higher in eWAT from *db/db* mice relative to *db*/+ mice (Figure 5(b)). Nucleotide-binding oligomerization (NOD) domain-like receptors (NLRs) are in the family of pattern recognition receptors and have been shown to regulate adipocyte differentiation *in vitro* [26]. We observed that expression of *NOD1* is suppressed in mice fed the WD (Figure 5(c)) and in *db/db* mice relative to lean controls (Figure 5(d)). *Ccl2*, a chemokine that recruits monocytes and macrophages, was more highly expressed in both WD-fed mice (Figure 5(e)) and in *db/db* mice (Figure 5(f)), relative to their respective lean controls. Moreover, transcript levels of *Cd68*, a macrophage marker, were elevated at 12 weeks by WD feeding (Figure 5(g)) and also enhanced in eWAT from *db/db* mice (Figure 5(h)).

### 3.5. There Is Elevated Expression of Glucose 6-Phosphatase, Acetyl Co-A Carboxylase, and Increased Acyl Glycerols in the Liver of *db*/*db* Mice

**Figure 6:**
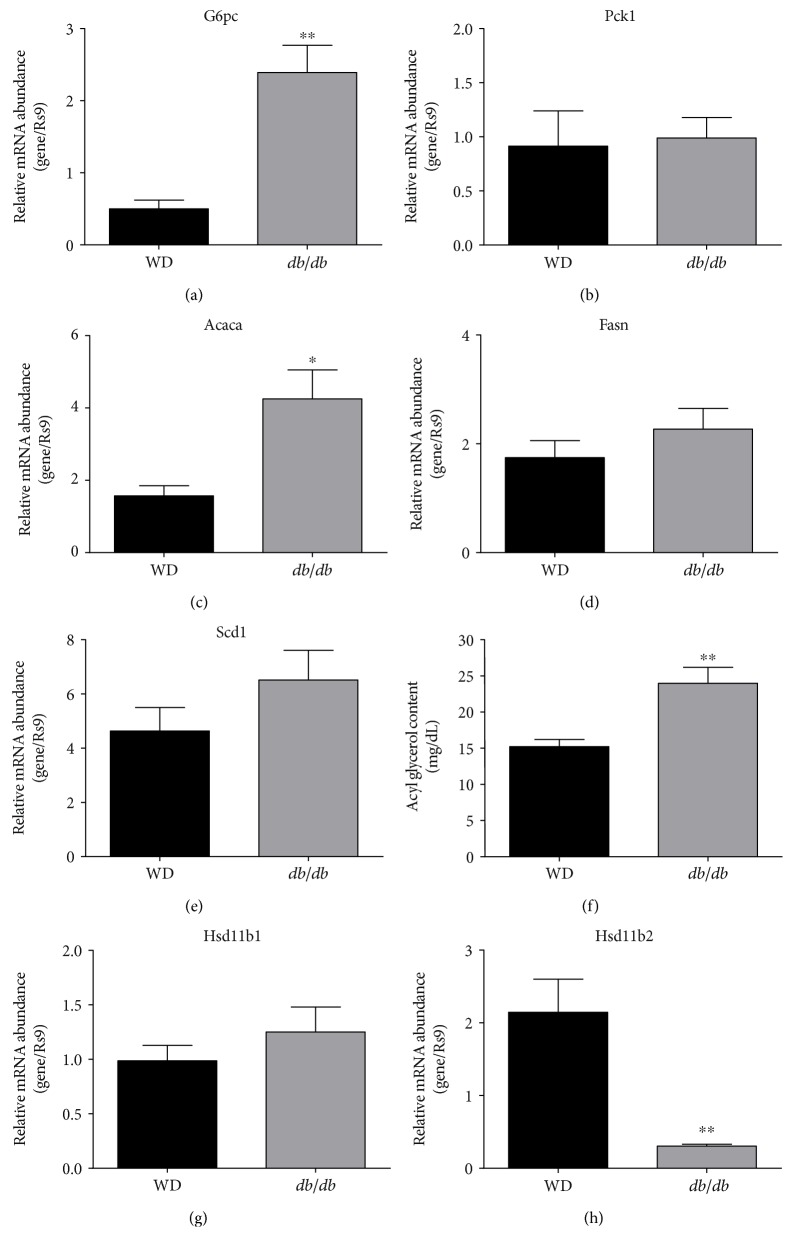
Livers from *db/db* mice displayed enhanced expression of glucose 6-phosphatase, acetyl Co-A carboxylase, and elevated acyl glycerol content when compared with livers from WD-fed mice. Relative mRNA abundance of the *G6pc* (a), *Pck1* (b), *Acaca* (c), *Fasn* (d), *Scd1* (e), *Hsd11b1* (g), and *Hsd11b2* (h) genes in the liver tissue from either C57BL/6J mice fed either Western diet for 20 weeks (black panels) or 14-week-old *db/db* mice (gray panels). Acyl glycerol content (f) was quantified using 30 mg of liver tissue from either Western diet-fed C57BL/6J mice (20 weeks on diet; black panels) or 14-week-old *db/db* mice (gray panels). *n* = 8 per group. Data are represented as means ± SEM. ^∗∗^*p* < 0.01; ^∗^*p* < 0.05.

Because severe insulin resistance in the liver is sufficient to create whole body glucose intolerance and elevations in blood glucose [27], we compared the expression of metabolic enzyme genes expressed in the livers of *db/db* mice and mice fed a WD. Livers from *db/db* mice displayed much higher expression of the gene encoding glucose 6-phosphatase (Figure 6(a)), while expression of Pck1 was similar between the obese mice (Figure 6(b)). Expression of acetyl co-A carboxylase 1 was 2.7-fold higher in *db/db* mice relative to mice fed a WD (Figure 6(c)), while expression of fatty acid synthase (Figure 6(d)) and stearoyl co-A desaturase 1 (Figure 6(e)) were similar between the mice. Consistent with the elevated levels of acetyl co-A carboxylase mRNA, we found that total liver acyl glycerols (mono-, di-, and tri-glycerols) were 57% higher in *db/db* mice relative to mice consuming the WD (Figure 6(f)). Relevant to corticosterone metabolism, the livers of WD-fed mice and *db/db* mice showed similar expression of the hsd11b1 gene, which encodes the enzyme 11beta-hydroxysteroid dehydrogenase 1 (Figure 6(g)). However, expression of the hsd11b2 gene was markedly reduced in *db/db* mice relative to mice fed the WD (Figure 6(h)).

### 3.6. There Is Reduced Skeletal Muscle Fat Oxidation in WD-Fed Mice, While Derangements in Both Skeletal Muscle and Liver Fat Oxidation Are Present in Weight-Matched *db*/*db* Mice

**Figure 7:**
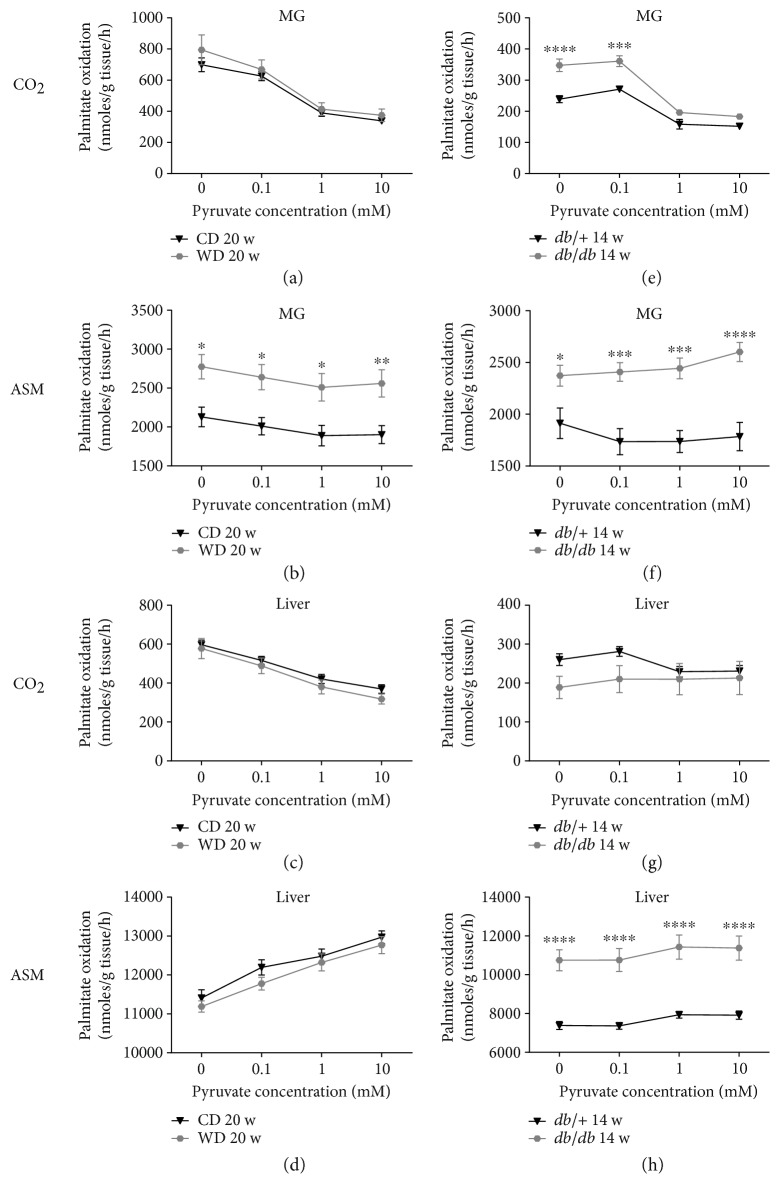
Derangements in the skeletal muscle and liver fat oxidation occur in *db/db* mice, while diet-induced obesity only negatively impacts skeletal muscle fat oxidation. Using [1-^14^C] palmitate (100 *μ*M), substrate switching was assessed by measuring complete (CO_2_) and incomplete (acid soluble metabolite (ASM)) fat oxidation ± varying doses of pyruvate as a competing substrate. Assays were run using homogenates from mixed gastrocnemius (MG) skeletal muscle (a, b, e, f) and liver (c, d, g, h) from diet-induced (control diet versus Western diet for 20 weeks; a–d) and a genetically obese model (*db/+* versus *db/db* mice aged 14 weeks; e–h) of obesity. *n* = 8 per group; means ± SEM. ^∗∗∗∗^*p* < 0.0001 versus control; ^∗∗∗^*p* < 0.001 versus control; ^∗∗^*p* < 0.01 versus control; ^∗^*p* < 0.05 versus control by two-way repeated measures ANOVA with Bonferroni post hoc test for multiple comparisons.

A trait observed during obesity-associated insulin resistance is the development of metabolic inflexibility, which is defined as the inability to appropriately transition between different fuel sources [28]. To test whether or not differences in metabolic flexibility occur at the tissue level in the diet-induced and genetic models of obesity, we used mixed gastrocnemius (MG) skeletal muscle and liver homogenates to measure fatty acid oxidation under conditions designed to test substrate selection (Figure 7). Compared to mice fed a control diet, MG from mice fed a Western diet had no difference in complete (CO_2_) palmitate oxidation (Figure 7(a)); however, MG from these mice did have elevated incomplete fatty acid oxidation rates (i.e., acid soluble metabolites (ASM); see Figure 7(b)). Alternatively, MG from *db/db* mice revealed higher baseline complete (Figure 7(e)) and incomplete (Figure 7(f)) palmitate oxidation than the lean *db/+* controls. Interestingly, the rates of CO_2_ production from both *db/+* and *db/db* mice are distinctly lower than C57BL6/J mice fed either a control or Western diet (Figure 7(a)). Importantly, palmitate oxidation rates (CO_2_ and ASM) in MG from both models of obesity had a main effect for pyruvate concentration indicating substrate switching; however, only genetically obese mice had an interaction effect (CO_2_*p* = 0.0054; ASM *p* = 0.0005), indicating *db/db* mice exhibit a different substrate switching effect than their lean *db/+* counterparts. Moreover, mice fed the control diet and Western diet (Figure 7(b)), as well as *db/+* mice (Figure 7(f)), all exhibit a decrease in ASM production as pyruvate concentrations increase. However, *db/db* mice have a contrasting rise in incomplete palmitate oxidation under the same conditions (Figure 7(f)).

In the liver, feeding a Western diet did not alter complete (Figure 7(c)) or incomplete (Figure 7(d)) palmitate oxidation. By contrast, the liver from *db/db* mice have slightly lower basal CO_2_ production (Figure 7(g)) and clearly demonstrate substantially exaggerated incomplete fat oxidation (Figure 7(h)). As pyruvate concentrations were increased, an interaction effect was observed for complete palmitate oxidation (*p* = 0.0048) indicating the *db/db* mice had virtually a complete lack of substrate switching compared to *db/+* controls (Figure 7(g)). Alternatively, no interaction effect was found concerning ASM production, indicating the impact of increasing pyruvate levels on incomplete hepatic fat oxidation was similar between *db/+* and *db/db* mice (Figure 7(h)). It is notable that *db/+* and *db/db* mice have lower complete hepatic palmitate oxidation rates, as well as reduced pyruvate-induced shift in hepatic fatty acid oxidation (CO_2_ and ASM) than C57BL6/J mice fed either a control or Western diet (Figure 7).

### 3.7. Circulating Insulin Levels Are Elevated in Both Models of Obesity, but Glucagon Is Only Increased in the *db*/*db* Mice

**Figure 8:**
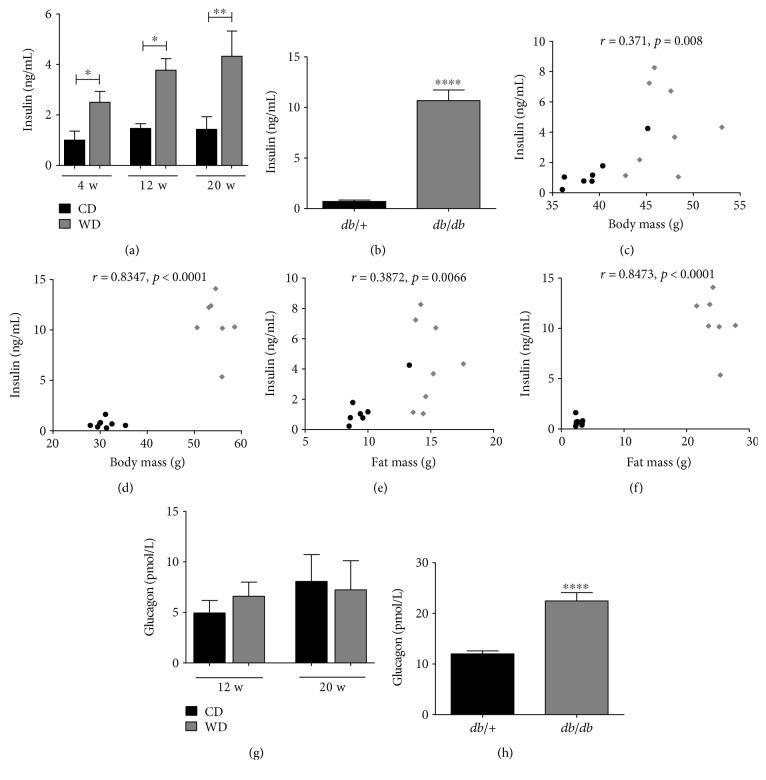
Circulating insulin levels are elevated in both mouse models of obesity, but glucagon is increased only in the *db/db* mice. Serum insulin (a, b) and glucagon (g, h) in C57BL/6J mice fed either control or Western diet for the indicated number of weeks (a, g) or 14-week-old *db*/+ and *db/db* mice (b, h). Serum insulin correlated to body mass or fat mass in C57BL/6J mice fed Western diet for 20 weeks (c and e, resp.) and in 14-week-old *db/db* mice (d and f, resp.) by Spearman's correlation. *n* = 8 per group; means ± SEM; ^∗∗∗∗^*p* < 0.0001; ^∗∗^*p* < 0.01; ^∗^*p* < 0.05. CD: control diet; WD: Western diet.

Hyperinsulinemia is present during obesity and is an independent risk factor for human disease [29, 30]. When analyzing this parameter in the obese mouse models, we found 2.5-, 2.6- and 3-fold increases in circulating levels of insulin at 4, 12, and 20 weeks, respectively, in WD-fed C57BL/6J mice compared to mice fed the control diet (Figure 8(a)). By comparison, *db/db* mice have 15.1-fold more insulin in circulation than their *db*/+ counterparts (Figure 8(b)) at 14 weeks of age. Serum insulin levels strongly correlated with both total body mass (Figures 8(c) and 8(d)) and fat mass (Figures 8(e) and 8(f)) in both diet-induced and genetic mouse models of obesity. In contrast to insulin, glucagon levels are unchanged in WD versus CD at 12 and 20 weeks of feeding (Figure 8(g)), while *db/db* mice displayed 87% more circulating glucagon when compared to the lean *db*/+ control mice (Figure 8(h)).

### 3.8. The Islets of *db*/*db* Mice, but Not Western Diet-Fed Mice, Display Markers of Dedifferentiation

**Figure 9:**
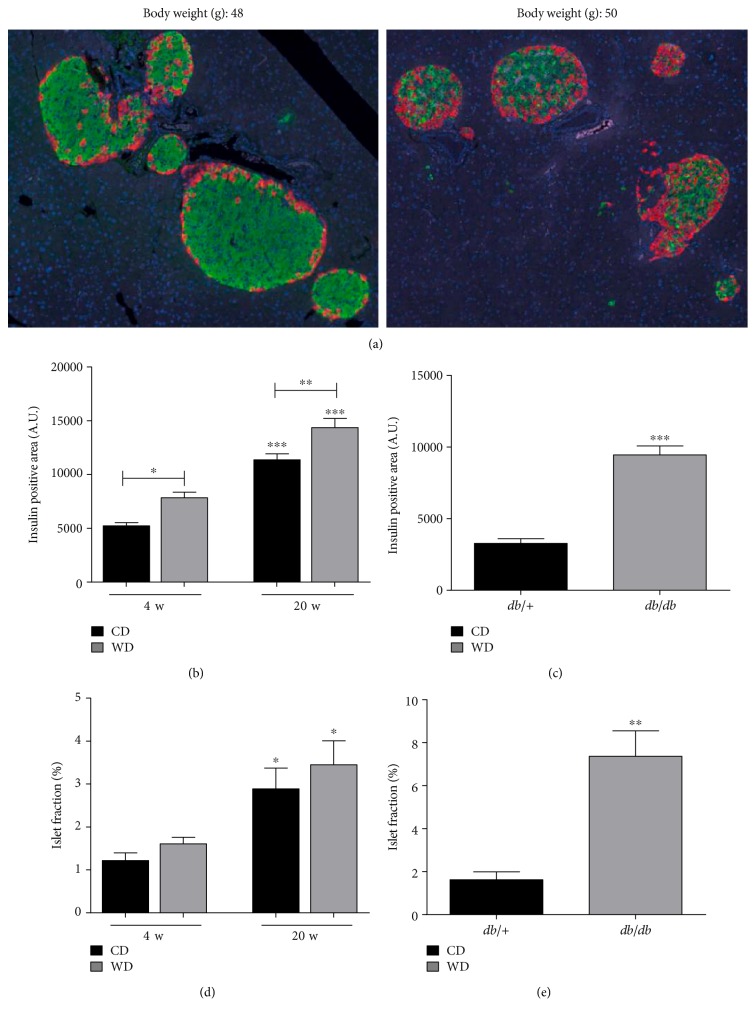
Islet size and insulin-positive area increase in both *db/db* and WD-fed mice. (a) Immunofluorescent images from pancreatic sections stained for insulin (green), glucagon (red), and DAPI nuclear stain (blue) from C57BL/6J fed WD for 20 weeks (left panel) or 14-week-old *db/db* mice (right panel). Insulin-positive area (b and c) and calculations of islet area relative to total pancreatic area, that is, islet fraction (d and e) are shown for C57BL/6J mice fed either control or Western diet for 4 or 20 weeks (b, d) and 14-week-old *db*/+ compared with *db/db* mice (c, e). Insulin-positive area and islet fraction were quantified using 8 mice per group. Data are shown as mean ± S.E.M. ^∗∗∗^*p* < 0.001; ^∗∗^*p* < 0.01; ^∗^*p* < 0.05. CD: control diet; WD: Western diet.

Insulin resistance drives increases in pancreatic islet *β*-cell mass [9]. Therefore, we next examined islet architecture between WD-fed mice and *db/db* mice. Both models of obesity display increases in insulin positive area over lean control mice (Figure 9()). However, islets from *db/db* mice display clear evidence of glucagon-positive cells within the core of the islet, which was not regularly observed in mice fed the WD (Figure 9(a)). This is consistent with an increase in glucagon transcripts within islets of *db/db* mice relative to WD-fed mice (not shown) and an increase in circulating glucagon in *db/db* mice (Figure 8(h)). In addition, we observed less immunoreactive Nkx6.1 in the nuclei of the *db/db* islets, which is consistent with pancreatic islets from humans with T2D [14]. By contrast, the islets of WD-fed mice retained strong evidence of nuclear Nkx6.1 expression (Figure 0()(a)). Furthermore, Aldh1a3 expression was markedly elevated in *db/db* islets when compared with islets from WD-fed mice (Figure 0()(b)). The increase in transcript is congruent with the increase in immunoreactive Adh1a3 in islets from *db/db* mice, but not in WD-fed C57BL/6J mice (Figure 0()(c)). Taken together, these data are consistent with the premise that WD-fed mice display features of insulin resistance (e.g., islet mass expansion), while islets from *db/db* mice display features of T2D (e.g., elevated glucagon and indicators of dedifferentiation).

## 4. Discussion

There are many important signs and symptoms that indicate risk for the eventual development of T2D, including obesity, inflammation, insulin resistance, and hyperinsulinemia [4, 5, 30, 31]. The use of mouse models is important for understanding the specific pathways contributing to alterations in tissue function, insulin sensitivity, islet hormone production, *β*-cell proliferation, insulin resistance, and overall glucose homeostasis. Therefore, we compared two distinct models of obesity in mice, the Western diet-fed C57BL/6J mouse and the *db/db* mouse on the C57BL/6J genetic background. The results presented in this study have led us to conclude that in mice with similar body weights, the WD-fed C57BL/6J mouse is an excellent model of early features representing the human prediabetic condition, while the *db/db* mouse more closely resembles human T2D.

Elevations in circulating insulin were evident within four weeks after introduction of the WD to mice (Figure 8(a)). These data are consistent with hyperinsulinemia in *db/db* mice (Figure 8(b)) and observations in the human population [32]. The present findings are important because hyperinsulinemia is a risk factor for many, if not all, symptoms used to denote the metabolic syndrome [33–35]. These conditions are readily recapitulated in the WD-fed mouse model and in the genetically obese *db/db* mice. In addition, elevated insulin levels have been suggested to be a causal factor for obesity [36, 37]. The idea that insulin influences body weight and overall adiposity is consistent with the correlations calculated for circulating insulin with total body mass (Figures 8(c) and 8(d)) and fat mass (Figures 8(e) and 8(f)) in both WD-fed and genetically obese *db/db* mice. Moreover, reducing insulin levels, by using diazoxide to decrease *β*-cell insulin output, restricts body weight in rodents by enhancing metabolic rate and increasing fat oxidation [38].

Human twin studies demonstrate that obesity has a significant genetic component [39]. In addition, there is evidence that islet *β*-cell failure, which is required for progression to T2D, also has a genetic basis [40]. Indeed, mutations in genes important for adult *β*-cell phenotype, such as GLUT2, glucokinase, NeuroD/beta2, HNF1/4, and Nkx6.1, contribute to deterioration of *β*-cell function in human T2D [41]. In our analyses of islets from mice fed a WD versus islets from *db/db* mice, we found that Nkx6.1 is reduced or absent from the nuclei of *β*-cells in the islets of *db/db* mice (Figure 9(a)). Nkx6.1 plays a key role in negatively controlling expression of the glucagon gene and therefore maintaining islet *β*-cells in a mature, fully differentiated state [15, 16].

The reduction in nuclear Nkx6.1 that we observe in *db/db* islets is also consistent with findings in pancreatic tissue from Zucker diabetic fatty rats [42] and in human subjects with T2D [14]. The increase in glucagon-positive cells we found in *db/db* islets (Figure 9(a)) connects changes in the pancreatic islets with the enhanced circulating glucagon in these mice (Figure 8(h)). Moreover, these data are congruent with reductions in Nkx6.1 transcripts (see [18]) and nuclear protein abundance in the islets of *db/db* mice (Figure 0(a)). Importantly, our findings are relevant to human disease because hyperglucagonemia is a key feature of the diabetic state [17, 43, 44]. Insulin resistance in pancreatic alpha cells may explain the elevations in circulating glucagon observed in *db/db* mice. Furthermore, deletion of glucagon receptors prevents hyperglycemia in obese mice with elevated glucagon [45]. We also see increases in Aldh1a3 transcript and protein in *db/db* islets (Figures 0(b) and 0(c)), a recently identified marker of dysfunctional and/or dedifferentiated *β*-cells [46]. Aldh1a3 is not elevated in the islets from WD-fed mice (Figures 0(b) and 0(c)) or any of the lean control mice (not shown), which supports our interpretation that *db/db* mice display phenotypes consistent with human T2D while WD-fed C57BL/6 mice may represent an earlier, nondiabetic stage of obesity and insulin resistance.

A metabolic signature of insulin-resistant skeletal muscle is an elevation in incomplete fatty acid oxidation rate, often measured as accumulation of acid soluble metabolites [22, 23]. Consistent with this notion, results from the present study show heightened ASM production in both diet-induced and genetic models of obesity. We further note that complete palmitate oxidation in *db/db* mice is higher than *db/+* controls in the absence of pyruvate; however, when moderate (1 mM) or high (10 mM) doses of pyruvate are present, CO_2_ production from palmitate is similar between skeletal muscle from *db/+* and *db/db* mice (Figure 7()). At first glance, these findings are striking as they suggest that metabolic flexibility at the level of the mitochondria may actually be better in *db/db* mice. However, this apparent improvement seems to come at the expense of an aberrant response of incomplete fatty acid oxidation (ASMs increase in presence of higher pyruvate concentrations), suggesting that greater mitochondrial overload may be occurring in the *db/db* mice.

In the liver, feeding a Western diet did not alter complete (Figure 7()(c)) or incomplete (Figure 7()(d)) palmitate oxidation. Also, while a main effect for pyruvate concentration was observed in liver, there was no interaction effect, suggesting that the Western diet had little impact on hepatic fatty acid oxidative capacity or substrate selection at the level of the mitochondria. It is, however, worth noting that as pyruvate concentration rises, the liver of the C57BL6/J mice in the WD-fed group responds by decreasing CO_2_ liberation and increasing ASM production, whereas the effect of pyruvate on hepatic fat oxidation in *db/+* and *db/db* mice is much less pronounced. Elevations in ASMs in response to increasing pyruvate levels could indicate a shift of carbons toward other metabolic outcomes (e.g., ketogenesis); however, the hyperinsulinemia induced in the diet-induced (WD fed) obese mice appears sufficient to maintain normoglycemia, which likely prevents any elevations in circulating ketones.

In adipose tissue, we detected increases in IL-1*α* (Figures 5(a) and 5(b)), a key cytokine that signals through the IL-1R1 and likely contributes to inflammatory responses relevant to insulin resistance. In addition, the expression of NOD1 was suppressed in both C57BL/6J mice fed the WD and *db/db* mice (Figures 5(c) and 5(d)). NOD1 activation blocks adipocyte differentiation *in vitro* [26], suggesting that this pattern recognition receptor may influence the degree of adiposity. We now provide *in vivo* evidence that NOD1 expression is decreased in two different mouse models of obesity, suggesting that reduced NOD1 expression is a component of adipose tissue expansion. By contrast, CCL2, a chemokine that attracts monocytes and macrophages, was elevated in adipose tissue of both WD-fed mice and *db/db* mice (Figures 5(e) and 5(f)). The CCL2 data are congruent with an increase in the macrophage marker CD68 (Figures 5(g) and 5(h)) and with macrophage accumulation within adipose tissue of obese rodents and humans [47–49].

We note that *db/db* mice display elevations in both glucagon and corticosterone (Figures 4() and 8()), which may explain their elevated blood glucose levels relative to mice made obese by WD feeding (Figure 1()). It is possible that *db/db* mice have reduced ability to properly regulate their hypothalamic-pituitary-adrenal axis due to deficiencies in leptin signaling, thus producing elevations in glucose and corticosterone. This interpretation is supported by studies from the Friedman group where specific deletion of the leptin receptor in hypothalamic brain regions results in elevated plasma levels of leptin, glucose, insulin, and corticosterone [50]. Thus, physiological leptin signaling appears to be required for proper corticosterone regulation, which is supported by additional studies [51]. In addition, we observed much less mRNA encoding for the 11-*β*-hydroxysteroid dehydrogenase 2 enzyme (Figure 6(h)). Since this enzyme is responsible for converting active steroid to inactive steroid, this observation could help to explain the elevations in circulating corticosterone or prolonged actions of the hormone in *db/db* mice. Finally, elevations in corticosterone and glucagon present in the *db/db* mice, but not the mice fed a WD, are consistent with the higher blood glucose observed in *db/db* mice.

In summary, we conducted a systematic analysis of two fundamental mouse models of obesity, one representing early stages of insulin resistance (WD fed) and the other displaying key features of overt T2D (*db/db*). With various pharmacologic, dietary, and exercise-based interventions being pursued to prevent or reverse the obese and insulin resistance state, our novel results are intended to provide a convenient resource for choosing a mouse model that best mimics the desired stage of human disease to be studied.

## Figures and Tables

**Figure 10:**
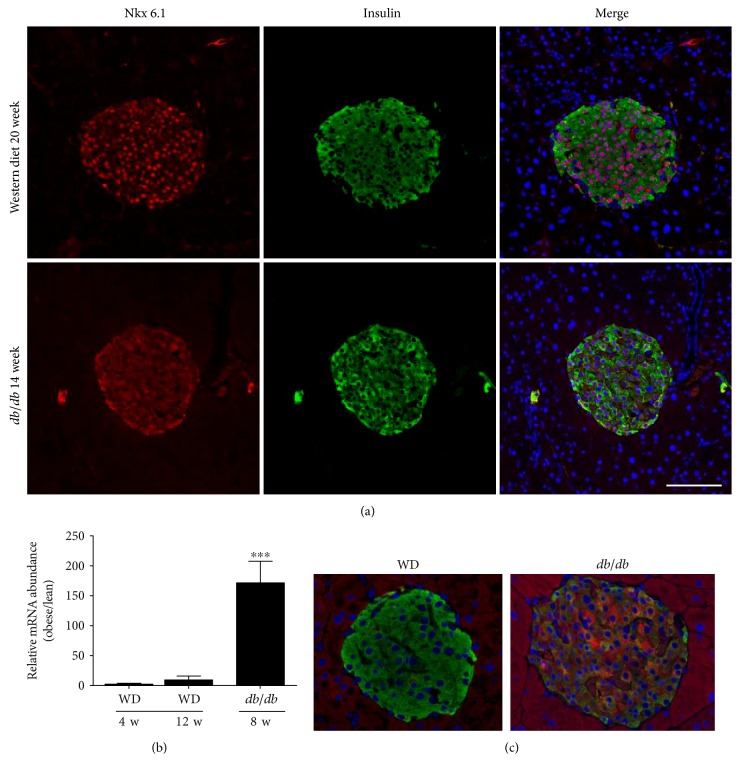
Markers of dedifferentiation are present in islets of *db/db*, but not weight-matched WD-fed mice. (a) Immunofluorescent analysis of islets from weight-matched WD-fed mice (top row) and *db/db* mice (bottom row) showing Nkx6.1 (red), insulin (green), and DAPI (blue). In the merged image, note the loss of double-positive nuclei (DAPI plus Nkx6.1; purple color) in the *db/db* mice but not in the WD-fed mice. (b) Expression of the Aldh1a3 gene in islets isolated from mice fed a WD for 4 or 12 weeks normalized to mice fed a control diet compared with *db/db* mice at 8 weeks of age (normalized to lean *db*/+ controls). ^∗∗∗^*p* < 0.001 versus both WD groups by one-way ANOVA. (c) Staining for Aldh1a3 protein (red) and insulin (green) in islets from weight-matched WD-fed mice (20 weeks on diet) versus *db/db* mice (14 weeks of age).
